# How to review a manuscript for journal publication: A primer for surgery residents

**DOI:** 10.1016/j.sopen.2024.12.007

**Published:** 2025-01-06

**Authors:** Nicholas J. Zyromski, David Stewart

**Affiliations:** a Indiana University School of Medicine, United States of America; b Southern Illinois University School of Medicine, United States of America

## 1 Introduction

Manuscript review for scientific publication is an important component of the academic process. Surgical residents entering a lab experience are in a good position to engage in manuscript review; however, few published guidelines to this process are available. The goal of this review is to present experience journal editors' thoughts on rationale and approach to the review process. The impetus was directed to surgical trainees; however, hopefully will be useful for reviewers of any experience.

## 2 Why review?

Some basic reasons to engage in the peer-review process include:

• citizenship
• recognition
• education*
• stewardship

A primary reason to engage in manuscript review is responsible academic citizenship. Surgeons publishing in academic journals rely on colleagues' peer review to inform and improve their manuscript and ultimately guide editors' decisions on publication. As such, responsible academic citizenship includes participation in the peer review process.

Of course, pure altruism is not the sole reason to review manuscripts. Academicians gain recognition in the eyes of both editors and authors when they review manuscripts. In addition, and perhaps most importantly, the peer review process of manuscript review is an education for the reviewer. The reviewer gains new ideas for their own research, is exposed to new approaches to asking a specific scientific question, insight into new statistical methodology, and more. Even in a pure double blinded manuscript review construct, after an editorial decision has been made the reviewer is privy to the comments of both the other reviewers and the editors. It is always informative to see how other educated and experienced scientists and clinicians view a similar work. Not infrequently, one will find other reviewers noticed positive and negative elements to the submission that as a reviewer you perhaps did not appreciate. Many reviewers have sharpened their eye for future reviews by reading the critiques of other reviewers.

As a final reason to serve as a reviewer, it is an unfortunate reality that scientific fraud exists. Reviewing manuscripts, like any other skill, is refined through practice and experience. Serving as a reviewer provides an opportunity to help keep the scientific record accurate and clean. This is especially important in terms of how the published record influences decisions related to research funding, both in terms of directing research proposals by investigators and in terms of evaluating their merit by grant reviewers. In some cases, career advancement and even employment are affected by what is published, and reviewers thus help serve as gatekeepers for what is counted as scientific literature.

## 3 How to get review invitations?

Three simple ways to engage in the review process are to publish, to contact journal editors directly, and for trainees and junior faculty, to ask your mentors to help with their reviews.

Publication is a first step towards receiving review invitations. As a researcher publishes in a field, they gain exposure to journal editors and are added to publishers' databases. Many publishers keep a database of authors' field of interest, and use these categories to find appropriate reviewers for similar manuscripts.

A new reviewer may always contact a journal editor directly; every editor will be happy to provide review opportunities to new reviewers for their journal. One caveat to this approach, of course, is when soliciting a review directly to be sure to provide a timely and high-quality product. This scenario provides an *ad hoc* reviewer with an opportunity to work towards a position on the editorial board for that journal, which is a respectable goal for a surgeon willing to devote uncompensated personal time to review scientific works.

Surgical societies are another excellent resource for entrance into manuscript review. A surgical society's recorder is responsible for managing the large bolus of manuscripts that accompany every society's meeting. Contacting the society recorder directly (perhaps even in person at the meeting) to introduce oneself and request a review is a highly effective method of soliciting reviews.

Finally, for trainees and junior faculty, using your mentors as a resource for introduction into the review process is also quite effective. This is helpful to all involved: it provides assistance to the faculty mentor, who may have multiple articles at a time to review within a typical 14–21 day time frame; it provides a young surgeon the opportunity to hone scientific and critical thinking skills from the perspective of a reviewer; and it helps the journal and the authors of the manuscript by providing more timely disposition to the submission.

## 4 How to review

Perhaps the most important goal of conducting a manuscript review is to remember that your primary job as a reviewer is to help the author improve their manuscript. This point bears repeating: *Your job as reviewer is to help the authors improve their manuscript*. Use the resources that are available to you – you may already be an expert in the subject material but if not, take a moment to review some of the manuscript authors' references or perform a quick literature search on the topic. Most journals have resources to support reviewers as well; these include free access to full text articles from the publisher's databases, reference search tools, and others.

Decisions about whether the manuscript topic falls within the scope of the journal and at the level of journal's quality are typically made by the editor prior to inviting review. However, these are good points of which to be aware.

Another important point to consider is novelty. Experienced authors will highlight the novelty of their work in the introduction and the discussion sections of their manuscript. It is worth reviewing references as well. This is a point where a very quick PubMed search will give you a perspective on the degree of novelty of the submission. As a reviewer, most of the submissions you will review will provide an incremental advance in scientific understanding, and as a reviewer, it is fair to evaluate why the submission should be published in light of whether the authors have identified a knowledge gap and whether the submission truly helps decrease that gap.

Is the work hypothesis driven research? Ideally all research questions start with a focused hypothesis and develop the methodology with which to answer this question. Sometimes this point is not obvious, but it is always useful to highlight. This aspect of manuscript review is incredibly important to interpreting the results of retrospective studies reviewing clinical data; there are circumstances where authors provide *statistically significant* results produced by post-hoc analysis of large, registry datasets. If these findings are not hypothesis directed, they are likely not reliable findings (despite “significant” *p* values listed alongside them).

Are the manuscript conclusions supported by the data presented? This is perhaps the single most important question to answer as a reviewer, and reviewers will find many submissions that provide conclusions extending well beyond what a reasonable interpretation of the data would justify. One does not need to be a subject or content expert to determine this point: all reviewers can evaluate the cogency of a manuscript.

Review the methodology. Is this basic science research? Does the study have appropriate positive and negative controls? Is the research question addressed with more than one methodology? For example, is there RNA message and protein or just one or the other used to support the hypothesis. Is the experiment done with *in vivo* methodology or simply *in silico* estimations? In the context of clinical research some questions to consider are the following: is this report a single institution series? Data should be quite granular in single institution series. Is it a prospective randomized trial? Case – control type series? Simple case series of descriptive data? All of the above have different levels of sophistication and therefore are more or less attractive to a journal depending on the scope of the journal, manuscript novelty and the specific research question.

Review the results with an eye towards tables and figures. Are the tables clearly legible? Tables typically represent the opportunity to present a large volume of data. Do the figures highlight important aspects of the author's message? Can the visual representation of data be streamlined or enhanced? Is variance represented clearly? Do the tables and figures support the conclusions or do they tell a different story and the narrative in the results section?

Statistics – the reviewer does not need to be an expert statistician; however, a general understanding of statistics will enhance anyone's ability to interpret the surgical literature. In the situation of very unusual or very sophisticated statistical analyses it is fair for a reviewer to point out that they are not familiar with these calculations and to thus suggest to the editors that the manuscript would benefit from review by a professional statistician. Please keep in mind, especially with the proliferation of large database driven papers, that very large sample size will yield statistically significant differences despite very small numerical differences. It is worth asking the question of how important the finding or difference is from a clinical perspective.

Discussion – it is helpful to review the discussion for length; in many cases, the discussion can both be shorter and more focused on explaining and commenting on the presented results. It is also important to see whether the authors have appropriately discussed their findings in relation to existing published literature. Every discussion should include a limitations paragraph as *every* publication has limitations. Reviewers should read this section of the discussion carefully to ensure that the limitations listed are not stock phrases but rather represent an insightful self-assessment of the work.

Language – this paper is published in *Surgery Open Science*, an English language journal. It is admirable to publish scientific work in a second language, but it is important that language be used properly. It is fair to point out to the authors when English syntax, grammar, usage, spelling, and punctuation could be improved. Every major publisher offers English language service (for example, Elsevier Author Services: https://webshop.elsevier.com).

## 5 Review presentation

No one universally preferred method for structuring manuscript reviews exists. We describe two frequently used approaches. In the first structure, a general summary of the manuscript is presented; editors who are managing multiple submissions greatly appreciate this summary. The summary is followed by major comments, minor comments, and a confidential note to the editors. The general summary may include such comments as assessments of novelty, clarity of presentation, and experimental design. Major comments, of course, are issues that the authors must fix before the manuscript is suitable for publication. Examples of major comments include requests for further detail regarding results, expanding upon experimental design, or highlighting the novelty of the work. Minor comments are just that – misspellings, points about data presentation, and so forth. Every editorial manager website has a text box that allows reviewers to provide confidential comments to the editor. These are often a very frank assessment of the reviewer's opinion regarding the manuscript's suitability for publication, communicated in a manner that would often be too blunt to be appropriately directed to the authors. This section can also be used for highlighting certain concerns that would escalate the emotional pique of the authors, such as concern regarding duplicate publication or fraudulent data. Editors greatly appreciate when reviewers both identify these potential concerns *and* when they first provide these concerns to editors for further investigation, helping to ensure careful vetting before discussion with the authors.

A second style of review follows the structure of the paper. After a brief summary, comments are made specifically based on the section: for example, the abstract, introduction, methods, *etc*. This structure does not prevent confidential comments to the editor.

It is important for reviewers to ensure that the study obtained institutional IRB approval, and if indicated, approval for animal use and informed consent of human subjects.

## 6 Summary

In summary, the manuscript review process is far more than an important contribution of faculty to their citizenship in the surgical community. Manuscript review provides continuing education to the reviewer throughout the course of their career, while serving, along with editors, as custodians over the accuracy and honesty of the literature that directs patient care and research funding decisions. Lastly, none of these functions preclude reviewers, and editors, from being constructive and kind in their comments to authors. This last comment is an opportunity to demonstrate empathy alongside academic rigor. Reviewing, and learning from other reviewers, is a uniquely rewarding form of continuing education.

